# Small RNA perspective of physical exercise-related improvement of male reproductive dysfunction due to obesity

**DOI:** 10.3389/fendo.2022.1038449

**Published:** 2022-12-02

**Authors:** Tingting Lin, Shuyu Zhang, Yuchuan Zhou, Ligang Wu, Xinmei Liu, Hefeng Huang

**Affiliations:** ^1^ The International Peace Maternity and Child Health Hospital, School of Medicine, Shanghai Jiao Tong University, Shanghai Key Laboratory of Embryo Original Diseases, Shanghai, China; ^2^ State Key Laboratory of Molecular Biology, Shanghai Key Laboratory of Molecular Andrology, Center for Excellence in Molecular Cell Science, Shanghai Institute of Biochemistry and Cell Biology, Chinese Academy of Sciences–University of Chinese Academy of Sciences, Shanghai, China; ^3^ Obstetrics and Gynecology Hospital, Institute of Reproduction and Development, Fudan University, Shanghai, China; ^4^ Research Units of Embryo Original Diseases, Chinese Academy of Medical Sciences, Shanghai, China; ^5^ Key Laboratory of Reproductive Genetics (Ministry of Education), Department of Reproductive Endocrinology, Women’s Hospital, Zhejiang University School of Medicine, Hangzhou, China

**Keywords:** male infertility, obesity, physical exercise, sncRNA, microRNA

## Abstract

**Purpose:**

To study whether physical exercise can effectively ameliorate obesity-induced abnormalities in male fertility and provide a new perspective on the role of small noncoding RNAs in spermatogenesis in obese male mice.

**Methods:**

In this study, four-week-old C57/Bl6 male mice were randomly allocated to receive a control diet, a high-fat diet or physical exercise intervention for 40 weeks. Purified round spermatids and spermatozoa were obtained after intervention. Sperm motility, concentration, the ability of the sperm to undergo capacitation and acrosome reaction were assessed. Small RNA sequencing was conducted on round spermatids and spermatozoa. The small noncoding RNAs expression pattern was systematically analyzed.

**Results:**

The spermatozoa concentration and percentage of motile spermatozoa, the capacitation and acrosome reaction, and the reproductive success rate, including mating success and pregnancy success, were decreased or delayed in the obesity group compared with controls. Physical exercise was able to restore the parameters to normal levels. Three microRNAs were consistently upregulated and 5 were downregulated in round spermatids and epididymal spermatozoa between the obesity and control groups.

**Conclusions:**

This report provides evidence that the adverse effects of obesity could be offset after physical exercise. small noncoding RNAs, especially microRNAs in germ cells, may play an important role in the effects of obesity and physical exercise on spermatozoa.

## Introduction

From 1975 to 2021, the obese male population tripled worldwide, and the proportion of obese men of childbearing age (20 to 40 years old) was approximately 11% (1, 2). Obviously, obesity is a complex disease of multifaceted etiology, with its own disabling capacities, pathophysiology and comorbidities. It remains a modifiable risk factor for diabetes mellitus, cardiovascular disease, obstructive pulmonary disease, arthritis, and cancer.

In parallel with rising male obesity, male sperm quality and fertility have declined in recent decades (3, 4). Despite considerable research efforts devoted to understanding the biology of obesity and energy balance, it has become obvious that, to date, our evolving scientific knowledge about the etiology of obesity has been of little help to evaluate the relationships between the obesity epidemic and spermatogenesis, sperm quality and fertility. A meta-analysis including data from 717 men revealed that, compared with men of normal weight, the odds ratio for oligozoospermia or azoospermia was 1.11 for overweight, 1.28 for obese and 2.04 for morbidly obese men (5). In addition, there is strong evidence of a negative relationship for testosterone, sex hormone binding globulin and free testosterone with increased body mass index (BMI) (6). Therefore, male obesity is considered to play an important role in the decline of male fertility (4).

The prescription of physical exercise has now been suggested to be an important component of obesity treatment. Although the effects of physical exercise on male fertility are uncertain, the positive effects of moderate exercise on spermatogenesis have been described by comparing sperm parameters in exercise and sedentary men (7); higher levels of FSH, LH and testosterone have been recognized in physically active subjects than in sedentary subjects. More recent reports have concluded that moderate training is associated with improvements in sperm DNA integrity and semen quality and with reduced expression of seminal markers of inflammation and oxidative stress (8–10). A randomized controlled trial including 200 obese men showed that physical exercise could significantly increase semen volume, semen concentration, semen mobility and the percentage of normal morphology (11). In rat testes, physical exercise effectively protected against the detrimental effect induced by obesity by downregulating stem cell factor, upregulating ghrelin and normalizing oxidative stress (12). In addition, physical exercise intervention improved testicular development in rats fed a high-fat diet by modulating KISS-1/GPR54 expression (13). Despite the painful progress made in the concept of improving obese male infertility through physical exercise, the role of physical exercise on infertility and spermatogenesis in obese men has not been adequately described from a holistic perspective.

Small noncoding RNAs (sncRNAs) are polymeric RNA molecules that are less than 200 nucleotides in length and are usually noncoding. They can be divided into several categories according to their origin, function and length, including microRNA (miRNA, 20-24 nt), PIWI-interacting RNA (piRNA, 29-30 nt), small interfering RNA (siRNA, 20-27 bp), small nucleolar RNA (snoRNA), small rDNA-derived RNA (srRNA) (10), tRNA-derived small RNA (tsRNA), and small nuclear RNA (snRNA), which is also commonly known as U-RNA. According to previous RNA-sequencing data, the small RNA population in mature mouse sperm is dominated by tRNA-derived small RNAs (tsRNAs), a smaller population of microRNAs (miRNAs) and PIWI-interacting RNAs, and an appreciable amount of ribosomal RNA (rRNA)-derived small RNAs (rsRNAs) (14). Many related mammalian studies have shown that sncRNAs may be involved in the intergenerational inheritance of environment-induced phenotypes. In a paternal mouse model given a high-fat diet, Chen et al. showed that a subset of sperm tsRNAs, mainly from 5′ transfer RNA halves and ranging in size from 30 to 34 nucleotides, exhibited changes in expression profiles (15). Furthermore, injecting sncRNAs from obese males’ sperm into healthy fertilized embryos can induce offspring to fully or partially mimic paternal phenotypes, including behavioral changes, obesity and glucose metabolism disorder (15–17). In addition, there is now compelling evidence to support that these sncRNAs, especially microRNAs, are known to interact with gene expression, chromatin remodeling and genome protection against transposition during spermatogenesis and seem to affect male fertility (18).

This study focused on whether physical exercise could effectively ameliorate high-fat diet-induced abnormalities in male fertility, investigated sncRNAs in spermatogenesis in obese mice from a new perspective, and identified certain sncRNAs as biologically active molecules that suggest male fertility.

## Materials and methods

### Animals and intervention

**Figure 1:**
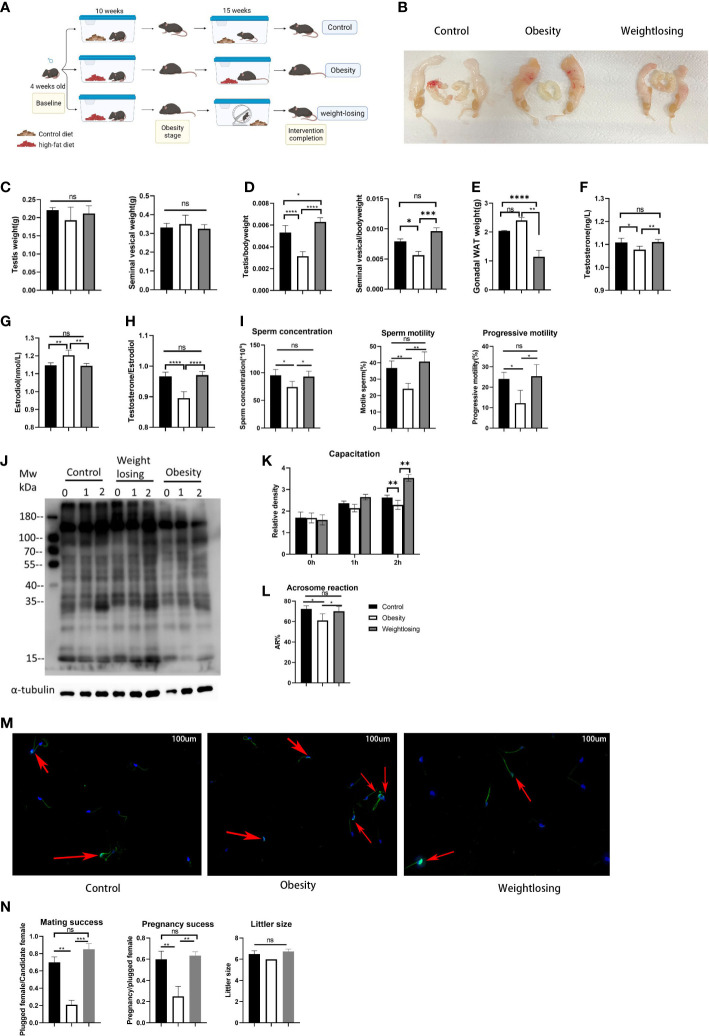
Effects of obesity and physical exercise intervention on reproductive morphology, reproductive hormones, sperm function and reproductive success. **(A)**, Schematic diagram of mouse breeding and intervention. **(B)**, Morphology of the mouse reproductive system. **(C)**, Testis weight (left) and testis index = testis weight/body weight (right). **(D)**, Seminal vesicle weight (left) and seminal vesicle index (right). **(E)**, Gonadal white adipose tissue weight. **(F)**, Serum testosterone (ng/L) levels. **(G)**, Serum estradiol (nm/L) levels. **(H)**, T/E2 and gonadal white adipose tissue weight (below). **(I)**, Analysis of semen traits include sperm concentration, motility and progressive motility. **(J)**, Western blot of sperm protein tyrosine phosphorylation to evaluate capacitation and its density. **(K)**, The relative density of the Western blot (p-tyrosine/α-tubulin). **(L)**, The proportion of sperm without intact acrosomes in the three groups. **(M)**. FITC-PNA was used to show the sperm acrosome and the sperm flagellum sheath. The red arrows indicate sperm with intact acrosomes that have not yet undergone acrosome reactions in the three groups. **(N)**. Mating success rate (left), pregnancy success (middle), litter size (right). One-way ANOVA was used to compare the differences among the three groups. Control: n=5, obesity: n=6, exercise: n=5. ns, P > 0.05, *P < 0.05, **P < 0.01, ***P < 0.001, ****P < 0.0001. Data are expressed as the mean ± SEM.

All mice were obtained from Shanghai Model Organisms, Shanghai, China. The animal ethics committee of Shanghai Model Organisms approved all experiments (Protocol Number: IACUC 2019-0016). All mice had free access to water and food and were maintained at the Shanghai Model Organisms animal house at 24 °C on a 12-h light, 12-h dark illumination cycle. Four-week-old C57BL/6 male mice were allocated into three groups. Group 1 (control, n = 5) received a control diet containing 4.3% fat, 19.2% protein and 67.3% carbohydrate (D12450B). Groups 2 (obesity, n=6) and 3 (exercise, n=5) received a high-fat diet providing 60% fat, 20% protein and 20% carbohydrate (D12492). Males were housed two or three per cage and maintained on these diet for 10 weeks until obesity model completion. The diet of Group 3 was then replaced with a control diet, and a running wheel with a lap counter was placed in the breeding cage. Males were housed two or three per cage and kept on these diet for 30 weeks until intervention completion (**Figure 1A**). Subsequently, each male was caged with four 7-week-old female rats for one week, and the vaginal plug was checked every day during this period. If the vaginal plug was found, mating success was confirmed, and the plugged female was moved to an individual cage. After 12 days, pregnancy success was confirmed. Littler size was counted on the delivery day. The reproduction experiment was carried out at 20 weeks and 30 weeks of physical exercise intervention. All experiments were performed with the evaluator blinded to the diet group and were performed by the same evaluator throughout the study.

### Body weight and body component measurements

Body weight for individual males was measured weekly. At the completion of intervention, body components were measured by the Body Composition Analyzer (QMR12-060H-I, Santan, Shanghai, China). Then, the mice were anesthetized with 1% pentobarbital sodium (50mg/kg body weight, intraperitoneal injection) for imaging with a magnetic resonance imager (NM21-060H-I, Santan, Shanghai, China). Then, the testes, seminal vesicles and gonadal white adipose tissue of the mice were separated and weighed.

### Serum hormones and lipids analyses

The mice were anesthetized, and their eyeballs were taken for blood. The serum was obtained by centrifugation at 1000 × g for 15 min after agglutination at room temperature for 1 hour. Testosterone and estradiol were measured by a mouse testosterone ELISA kit and a mouse estradiol ELISA kit according to the manufacturer’s instructions (Hu21207, Hu20201, BioTSZ, US). Serum cholesterol, triglycerides, high-density lipoprotein cholesterol, low-density lipoprotein cholesterol, and non-esterified fatty acids were measured by an automatic biochemical analyzer (Toshiba120, Japan).

### Glucose tolerance test and insulin tolerance test

For GTT, mice were fasted for 16 h (1700 to 0900 h) with free access to drinking water. A baseline blood sample was collected from the tails of fully conscious mice, followed by an intraperitoneal injection of glucose (0.75 g glucose/kg body weight), and blood was taken from the tails for glucose measurements at 0, 15, 30, 60, and 120 min. For insulin tolerance tests, mice were fasted for 6 h (0900 to 1500 h), and baseline blood samples were collected from the tails of fully conscious mice. Insulin (1 unit/kg body weight) (Humulin; Eli Lilly and Company, Indianapolis, IN, US) was administered by intraperitoneal injection, and blood samples were taken from the tail at 0, 30, 60, 90 and 120 min postinjection. Blood was collected from the retroorbital sinus after an overnight fast (16 h) to measure plasma insulin levels using a mouse ELISA kit (Crystal Chem Inc.).

### CASA for semen analysis

Computer-aided semen analysis (CASA) was performed using the Hamilton-Thorne Research IVOS sperm motility analysis system and the previously described version 10 software (Hamilton-Thorne, Danvers, MA) (19). CASA was performed on the epididymal semen sample placed in a Leja standard counting fixed cover glass slide (Leja Products B.V., Nieuw-Vennep, The Netherlands) with a depth of 20 microns. Using Olympus “negative phase” optics, the analysis is limited to 15-100 tracking points at a 60 Hz frame rate. The standard kinematics are calculated by the CASA program. Cells exhibiting an average path velocity of less than 10 μm·s-1 are considered nonmoving.

### Western blot of protein tyrosine phosphorylation

After male mice were anesthetized, their epididymal tails were removed, cut into pieces with ophthalmic scissors, and placed in PBS. This liquid was placed in a 37°C incubator for 10 min for the spermatozoa to swim up. Using a large pipette, the liquid containing the spermatozoa was aspirated and placed in a centrifuge tube. After centrifugation at 1000 × g for 1 minute, the supernatant was aspirated, an appropriate amount of PBS was added to mix, and the sample was centrifuged again at the same speed and time as before. Washed spermatozoa were diluted with 1 ml of BWW (Solarbio, G2585) and quickly divided into three equal parts. One part was quickly added to the protein denaturation solution (Tris-HCl 0.0625 M, SDS 2%, glycerin 10%, β-mercaptoethanol 5%) and heated for denaturation, and the other two parts were incubated at 37°C for 60 min and 120 min, respectively, to achieve sperm capacitation. After incubation, the other two parts were centrifuged as before, and the upper layer was aspirated. Then, protein denaturation solution was added and heated to denature. Then, the samples were separated by SDS‐PAGE and transferred to a PVDF (polyvinylidene fluoride) membrane. Next, the membranes were blocked with 5% skimmed milk powder and separately probed with rabbit anti Phospho-Tyrosine (8954, CST, USA) and rabbit anti α-tubulin (10094-1-AP, Proteintech, Wuhan, China) overnight at 4°C with a final dilution of 1:1000 in 5% milk. After three washes, the membranes were incubated with goat anti-rabbit secondary antibodies (SA00001-2, Proteintech, Wuhan, China) at a 1:2000 dilution at 37°C for 1.5 h. Images of the membranes treated with ECL (enhanced chemiluminescence) were captured by a Western Blotting Detection System (Tiangen, Beijing, China).

### Acrosome reaction by immunofluorescence

After the male mice were anesthetized, their epididymal tails were removed, cut into pieces with ophthalmic scissors, and placed in PBS. The liquid was placed in a 37°C incubator for 10 min for the spermatozoa to swim up. Using a large pipette, the liquid containing the spermatozoa was aspirated and placed in a centrifuge tube. After centrifugation at 1000 × g for 1 minute, the supernatant was aspirated, an appropriate amount of PBS was added to mix and wash the spermatozoa, and the samples were centrifuged again at the same speed and time as before. Washed spermatozoa were diluted with PBS to adjust the final sperm concentration to 1×10^4^ spermatozoa/ml. Add 20 µl of the diluted liquid to each smear so that the number of sperm on each smear is 200. Then, the cells were fixed with 4% PFA and stained with 0.5 mM PNA-FITC (L7381, Sigma‐Aldrich) and DAPI (D9542, Sigma‐Aldrich) as previously described. The stained spermatozoa were then covered with a cover slip (24 × 50 mm) (20).

### Acquisition of spermatids at different stages

As in previous research (21), the testes of the anesthetized mice were collected in PBS and placed on ice. After removing the tunica albuginea, the testis was placed in 5 mL of PBS containing 120 U/mL type I collagenase preheated at 37°C and gently stirred for 5 minutes. The dispersed seminiferous tubules were further digested with 5 mL 0.25% trypsin and 0.1 mL DNase I (5 mg/mL), gently pipetted several times at 37°C for 8 minutes, and then stopped by adding 0.5 mL fetal bovine serum (FBS) to inactivate trypsin. After the two-step enzyme digestion, the separated testicular cell suspension was filtered through a PBS prewet cell filter with a pore size of 70 μm. The cell suspension was centrifuged at 500 × g for 5 minutes at 4°C, and the supernatant was carefully removed from the pellet. The cells in the pellet were resuspended in DMEM containing Hoechst 33342 (3 mg/mL) and 5 μl DNase I at a concentration of 1 × 10^6^ cells/mL. The cell suspension was rotated at 10 r.p.m./min for 20 minutes at room temperature, centrifuged at 500×g for 5 minutes at 4°C, and resuspended in 0.3-1 mL DMEM for sorting. The cell population was collected based on the fluorescent label stained with Hoechst 33342 by fluorescence-activated cell sorting (FACS).

### Small RNA-seq

The RNAs of round spermatids and spermatozoa were extracted using the miRNeasy Serum/Plasma Kit (217184, Qiagen, Germany) according to the manufacturer’s protocol. The quantity and quality of RNA were assessed using a spectrophotometer (Nanodrop 2000, Thermo).

In this experiment, the single-end 50 bp sequencing mode of the Illumina HiSeq platform was used for high-throughput sequencing of samples. The original data were removed by primers and adaptor sequences, and the sequenced fragments with reliable quality were selected after inspecting sequence quality and length. Then, the type (unique) and quantity (total) of small RNA (sRNA) were counted, and length distribution analysis of small RNA was performed. We used fastx_Clipper to remove the splice sequence from the original data, filter out the low-quality sequence and select the sequence with a length of 14-40 nt for downstream analysis. Fastqc was used to evaluate the quality of the sequence, including mass density distribution, length distribution, redundancy statistics, etc. For all sequences after pretreatment and for the unique sequence after the duplication of each sample, Bowtie software was used for comparison with the reference genome, Rfam sequence database, RepBase sequence database and miRBase database of the species. The sequence alignment wass set to allow only one mismatch, and the comparison were statistically analyzed. The reads of each sample were compared to the existing miRNA database (miRBase) and the results of the new miRNA prediction to calculate miRNA expression (miRNA expression calculation uses CPM, counts per million). The meaning of CPM is to use the paired sequence per million ratio as an indicator of miRNA expression, where the total ratio of paired reads is used to normalize the expression value. DESeq software was used to analyze the differential expression of the Control and Case sample groups, screen the differentially expressed miRNAs, calculate the expression level of each sample and the intragroup mean, calculate the fold change difference between the groups, and then calculate log2(fold change) for subsequent use. Screening for differentially expressed genes, when p value <= 0.05 and log2(fold change)> = 1, we believe that such genes are significantly different between groups. The miRanda database was used to perform target prediction on differentially expressed miRNAs.

### GO annotation and KEGG pathway enrichment analysis

Gene Ontology (GO) annotation and Kyoto Encyclopedia of Genes and Genomes (KEGG) pathway enrichment analysis were conducted to investigate the roles of target genes of consistently differentially expressed miRNAs. GO analysis was performed to assess the biological implications of the target genes (22). We downloaded the GO annotations from NCBI (http://www.ncbi.nlm.nih.gov/), UniProt (http://www.UniProt.org/), and GO (http://www.geneontology.org/). Pathway analysis was performed to explore the significant pathways of target genes based on the KEGG database (23). The significant GO categories and KEGG terms were identified using Fisher’s exact test. The threshold of significance was defined by a P value < 0.01 for GO analysis and a P value < 0.05 for KEGG analysis. The GO-Tree is a directed acyclic graph, and each term has defined relationships to one or more terms (24). The mutual regulation relationships between enriched KEGG pathways were illustrated by pathway-act networks (25).

### Real-time quantitative PCR

First-strand cDNAs of small RNAs from round spermatids and spermatozoa were synthesized by a Bulge-Loop miRNA qRT‐PCR Starter Kit (C10211, RIBOBIO, Guangzhou, China) according to the manufacturer’s instructions. qPCR was carried out by using SYBR Green PCR Master Mix (TaKaRa, Dalian, China) and a LightCycler 480II Real-Time PCR System. The forward primers of small RNAs and universal reverse primers were designed based on the mature sequences of small RNAs (**Supplementary Table 1**). The expression levels of small RNAs were normalized with 5S to obtain the relative expression by the comparative CT method.

### Statistical analysis

We adopted α=0.05 (both sides) and β=0.20 as standard values, the proportion of Obesity Group and Control Group is 1:1 following the formula for comparison of two means to calculate the sample size (26). Normality of the data was tested using the Shapiro-Wilk normality test. Nonparametric data with multiple comparisons were analyzed by Kruskal-Wallis one-way analysis of variance followed by Holm’s Stepdown Bonferroni and non-paired analysis procedure for adjusted p-values. Data with normal distribution were analyzed by one-way ANOVA with Dunnett’s post-test or Tukey’s correction for multiple comparisons. Statistical analysis was conducted by GraphPad Prism V8.0 (GraphPad Software, San Diego, California, USA). All values are presented as the mean ± SD, and P values less than 0.05 (*), 0.01 (**), 0.001 (***), and 0.0001 (****) were considered significant differences.

## Results

### Effects of physical exercise on the reproductive system in obese mice

Male mice fed a high-fat diet for 10 weeks showed significant weight gain (obesity stage: control = 29.6 g vs. obesity = 36.6 g vs. exercise = 37.8 g, P<0.001), which means that the obese male mouse model was successfully constructed (**Supplementary Figure 1A**). After that, the exercise group began to perform physical exercise using an autonomous running wheel, and the obesity group continued to be fed a high-fat diet. At the end of 30 weeks of intervention, the weight of the mice in the exercise group was far lower than that of the obesity group and even significantly lower than the weight of the control group (control = 42.0 g vs. obesity = 61.0 g vs. exercise = 33.6 g, P<0.0001) (**Supplementary Figure 1A**).

When evaluating the effect of obesity and physical exercise intervention on reproductive organ morphology, we found that the testes and seminal vesicles of obese male mice were clearly reduced in volume relative to the overall reproductive system (**Figure 1B**). Then, we weighed the testes and seminal vesicles and found that the weights of the testes and seminal vesicles were not significantly different among the three groups (testis: control = 0.22 g vs. obesity = 0.19 g vs. exercise = 0.21 g, P=0.23; seminal vesicles: control = 0.33 g vs. obesity = 0.35 g vs. exercise = 0.32 g, P=0.87) (**Figure 1C**). However, when the weight of the testes and seminal vesicles was normalized for body weight, it was found that the relative weight of the testes or seminal vesicles was significantly reduced in the obesity group, and physical exercise intervention could restore it nearly to the control level (testis index: control = 0.5% vs. obesity = 0.3% vs. exercise = 0.6%, P<0.0001; seminal vesicles index: control = 0.8% vs. obesity = 0.6% vs. exercise =. 1.0%, P<0.001) (**Figure 1D**). These results indicated that obesity or exercise did not change the morphology of the reproductive organs themselves but affected the relative proportion of reproductive organs in the body.

To evaluate the effects of obesity and physical exercise intervention on sex hormones, we measured the concentrations of testosterone and estradiol in peripheral blood serum. Obese male mice had significantly lower testosterone levels and higher estradiol levels than control mice, and physical exercise intervention could restore the serum reproductive hormones (testosterone: control = 1.11 vs. obesity = 1.08 vs. exercise = 1.11, P<0.01; estradiol: control = 1.15 vs. obesity = 1.20 vs. exercise = 1.14, P<0.001) (**Figures 1F, G**). The ratio of testosterone to estradiol represents aromatase activity; the lower the ratio is, the stronger the aromatase activity. Obesity significantly reduced this ratio in male mice, while physical exercise restored it to the control level (control = 0.97 vs. obesity = 0.90 vs. exercise = 0.97, P<0.0001) (**Figure 1H**). In addition, gonadal white adipose tissue is rich in aromatase P450, the weight of which also reflects the potential activity of aromatase in male mice. The weight of gonadal white adipose tissue was significantly increased in the obesity group compared with the control group but significantly decreased in the exercise group (control = 2.04 vs. obesity = 2.40 vs. exercise = 1.15, P<0.0001) (**Figure 1E**). Not surprisingly, physical exercise could significantly reduce gonadal white adipose tissue accumulation due to obesity. These results suggested that physical exercise could effectively restore the sex hormone disorder caused by obesity by reducing the stock of aromatase.

To evaluate the effects of obesity and physical exercise intervention on sperm function, we used CASA to analyze semen traits. The sperm concentration was significantly lower in the obesity group than in the control (almost 78% lower than the control), while physical exercise restored it to normal levels (control = 95.3*10^6^/ml vs. obesity = 74.1*10^6^/ml vs. exercise = 93.0*10^6^/ml, P=0.01). In addition, the obesity group demonstrated a notably lower percentage of motile spermatozoa than the control group, mainly due to the decrease in spermatozoa with progressive motility. The exercise group showed a similar concentration of motile and progressive motile spermatozoa as the control group (motility: control = 36.8% vs. obesity = 24.2% vs. exercise = 40.8%, P=0.001; progressive motility: control = 24.0% vs. obesity = 12.2%vs. exercise =25.4%, P=0.01), which indicated that physical exercise could increase sperm concentration and improve sperm motility in obese males back to normal levels (**Figure 1I**).

In mammalian species, the acquisition of sperm fertilization competence is dependent on sperm capacitation. One of the key elements of capacitation is protein tyrosine phosphorylation (TP) in various regions of the sperm membrane. To evaluate the capacitation of spermatozoa, we detected tyrosine phosphorylation by western blotting. Sperm from the tail of the epididymis of mice were incubated for 0, 1 and 2 hours in complete culture medium supporting capacitation. Then, the proteins in sperm were extracted immediately, separated by SDS‐PAGE electrophoresis and incubated with tyrosine phosphorylated antibody. This experiment was carried out five times with similar results. The most representative figure is shown in **Figure 1J**; the other figures were detected density and are shown in **Figure 1K**. The process of sperm capacitation in obese male mice was slower, which was reflected in the lower tyrosine phosphorylation level at 1 hour of incubation than that in the control group. After incubation for 2 hours, tyrosine phosphorylation differed even more from that of the control group. However, the tyrosine phosphorylation of the exercise group was similar to that of the control group. This suggested that obesity could hinder sperm capacitation, while physical exercise in obese men could restore sperm capacitation.

To further evaluate the sperm acrosome reaction, we detected the retention of intact sperm acrosomes by immunofluorescence. The sperm were removed from the tail of the epididymis and washed twice with PBS. The washed sperm were diluted with PBS and fixed on the smear with paraformaldehyde so that there were approximately 200 sperm on each smear.The acrosome and flagellum sheath were labeled green, and the nucleus was stained blue under a fluorescence microscope after incubation with PNA-FITC and DAPI, respectively. **Figure 1M** shows the most representative figure. Compared with the control group, the sperm intact acrosome retention rate of obese male mice was higher, so the incidence of acrosome reaction was lower. The exercise group showed an intact acrosome retention rate and acrosome reaction similar to those of the control group (rate of acrosome reaction: control = 72.3% vs. obesity = 61.1% vs. exercise = 70.2%, P=0.01) (**Figure 1L**). This indicated that spermatozoa in obese male mice showed a delayed acrosome reaction, while physical exercise could restore it.

To evaluate the effects of obesity and physical exercise intervention on reproductive success, we mated each male mouse in the three groups with four 7-week-old female mice for one week at 20 and 30 weeks of intervention and checked vaginal plugs every day to determine mating success. For successfully mated females, pregnancy was confirmed at Day 12 after mating. Finally, the number of pups per litter was counted on the first day of delivery. The results showed a negative overall effect of obesity on mating and fertilization success, but physical exercise could still restore impaired reproductive success (**Figure 1N**).

To demonstrate whether the alteration of the reproductive system is associated with body composition and metabolism, we compared several parameters between the three groups. Through the analysis of the body composition of mice, it was found that the body fat ratio of obese mice increased significantly (obesity = 45.1% vs. control = 28.5%, P=0.001), mainly due to the accumulation of visceral fat. The red area that represents fat was significantly concentrated in the central area of the internal organs in the MRI imaging; this area significantly decreased after physical exercise intervention (exercise = 13.4% vs. obesity = 45.1%) (**Supplementary Figures 1B, C**). In addition, we found that the obese mice were glucose intolerant and insulin resistant, showing raised blood glucose at fasting and during a glucose tolerance test (**Supplementary Figure 1D**) and elevated serum insulin at fasting (**Supplementary Figure 1F**). The insulin tolerance test response was blunted (**Supplementary Figure 1E**). As expected, mice that had experienced physical exercise showed improvement in glucose intolerance and insulin resistance in their GTT and ITT (**Supplementary Figures 1D–F**). Obese mice showed impaired lipid metabolism—specifically, elevated serum total cholesterol (**Supplementary Figure 1G**), triglycerides (**Supplementary Figure 1H**), low-density lipoprotein (**Supplementary Figure 1J**), and non-esterified fatty acids (**Supplementary Figure 1K**), except high-density lipoprotein (**Supplementary Figure 1I**). Physical exercise intervention effectively reduced the blood lipids of obese mice and returned them to control level (**Supplementary Figures 1G–K**).

### sncRNA dynamics in mouse round spermatids and epididymal spermatozoa

**Figure 2:**
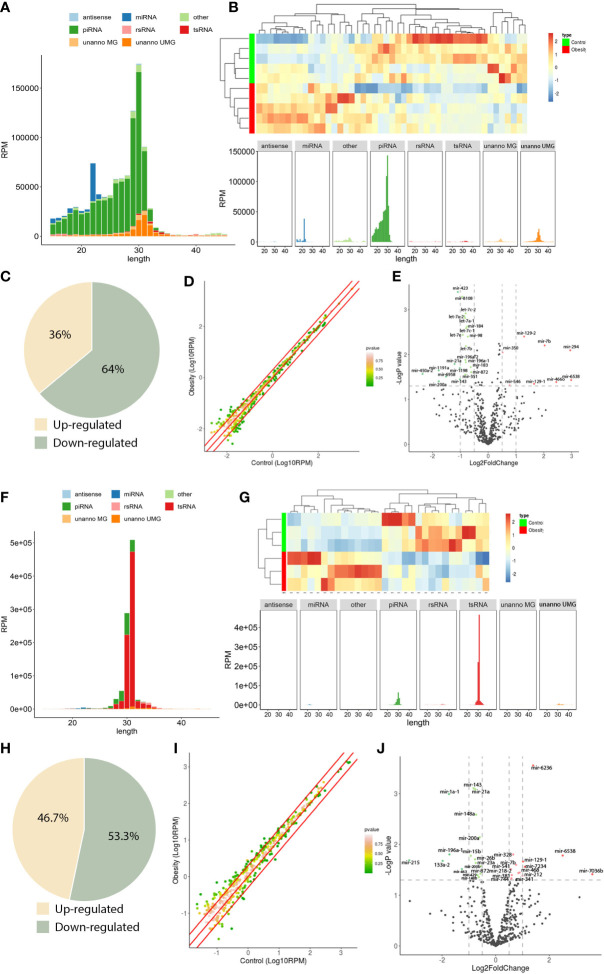
sncRNA dynamics in mouse round spermatids and epididymal spermatozoa. **(A)**, sncRNA length distribution in round spermatids. **(B)**, Heatmap of significantly differentially expressed miRNAs in round spermatids. Upregulated miRNAs are marked red, and downregulated miRNAs are marked blue. **(C)**, Scatterplot of miRNA expression variation between the obesity and control groups in round spermatids. The normalized expression values of each gene are shown on the x-axis and the y-axis. The miRNAs above the top red line or below the bottom red line had more than a twofold difference in expression between the two groups. **(D)**, The expression of a cohort of miRNAs in round spermatids from the obesity group differed from that in the control group. Of the 47 differentially expressed miRNAs, 17 were upregulated, and 30 were downregulated. **(E)**, Volcano plots of miRNA expression variation between the two groups in round spermatids. Red and green dots indicate significantly upregulated and downregulated miRNAs in the obesity group, respectively (P < 0.01). Gray dots represent nondifferentially expressed genes between the two groups. **(F)**, Length distribution of sncRNAs in epididymal spermatozoa. **(G)**, Heatmap of significantly differentially expressed miRNAs in epididymal spermatozoa. Upregulated miRNAs are marked red, and downregulated miRNAs are marked blue. **(H)**, Scatterplot of miRNA expression variation in epididymal spermatozoa between the obesity and control groups. The normalized expression values of each gene are shown on the x-axis and the y-axis. The miRNAs above the top red line or below the bottom red line had more than a twofold difference in expression between the two groups. **(I)**, The expression of a cohort of miRNAs in epididymal spermatozoa from the obesity group differed from that in the control group. Of the 30 differentially expressed miRNAs, 14 were upregulated, and 16 were downregulated. **(J)**, Volcano plots of miRNA expression variation between the two groups in epididymal spermatozoa. Red and green dots indicate significantly upregulated and downregulated miRNAs in the obesity group, respectively (P < 0.01). Gray dots represent nondifferentially expressed genes between the two groups.

To uncover the expression profiles of sncRNAs in mouse spermatogenic cells, we collected round spermatids (haploid, RS), spermatocytes (tetraploid), Sertoli cells and Leydig cells (diploid), and elongated spermatids (haploid, ES) from the testis by flow cytometry and epididymal spermatozoa (SP) by the swimming-up method. After setting a gate by forward scatter/side scatter characteristics and DAPI characteristics, round spermatids, elongated spermatids and spermatocytes were isolated, accounting for 18.6%, 1.3% and 6.2% of the testicular cells, respectively (**Supplementary Figure 2A**). To determine the purity of the isolated RSs, the expression of the RS marker *PRM1* (27) was examined by qPCR. Analysis showed that *PRM1* was significantly highly expressed in the isolated round spermatids (**Supplementary Figure 2B**). Then, total RNA was extracted from RS and SP, and small RNA-seq was conducted. The expression levels of sncRNAs in RS and SP were compared. The distributions of the total expression of sncRNAs between RS and SP were different, as illustrated by the barplot analysis (**Figure 2A**).

The majority of sncRNAs in RS were miRNAs and piRNAs, which accounted for 6.0% and 72.8% of the total sncRNAs, respectively (**Figure 2A**). However, in SP, the proportion of miRNA and piRNA decreased, accounting for 0.4% and 9.1%, respectively, and the proportion of tsRNA increased significantly, accounting for 85.5% (**Figure 2F**), indicating that the sncRNAs underwent dramatic changes during sperm maturation.

To further explore the function of sncRNAs in male fertility, we focused on the analysis of miRNA changes. First, we compared the expression of miRNAs in RS from the obesity and control groups. Systematic variations in the expression of miRNAs among samples were visualized by heatmap clustering analysis (**Figure 2B**). A scatterplot was used to evaluate the variation in miRNA expression between the obesity and control groups (**Figure 2C**). The threshold for differential expression was set to a P value < 0.05. In total, we identified 47 significantly differentially expressed miRNAs in RS: 17 were upregulated, while 30 were downregulated (**Figure 2D**). A volcano plot was constructed to identify significantly upregulated and downregulated miRNAs in the obesity RS group relative to the control group (**Figure 2E**). Then, we compared the expression of miRNAs in SP from the obesity and control groups. The heatmap clustering analysis showed the systematic variations in the expression of miRNAs among samples (**Figure 2G**). The scatterplot showed the variation in miRNA expression between the obesity and control groups (**Figure 2H**). In total, we identified 30 significantly differentially expressed miRNAs in SP: 14 were upregulated, while 16 were downregulated (**Figure 2I**). A volcano plot was constructed to identify significantly upregulated and downregulated miRNAs in the obesity SP group relative to the control group (**Figure 2J**). The data suggest that the expression of miRNAs in RS and SP cells from the obesity group differed from controls.

### GO analysis and KEGG pathway analysis of target genes of consistently differentially expressed miRNAs revealed significantly enriched biological process GO terms and signaling pathways

According to the above analysis, we found that there were 3 miRNAs (miR-6538, miR-129-1, and miR-7b) that were consistently upregulated in RS and SP. Five miRNAs (miR-196a-1, miR-872, miR-21a, miR-143, and miR-200a) were consistently downregulated in the RS and SP groups compared with the obesity and control groups. MicroRNAs have been extensively reported to execute their functions by binding to their targets. miRNA targets were predicted by miRanda and were considered potential miRNA targets.

**Figure 3:**
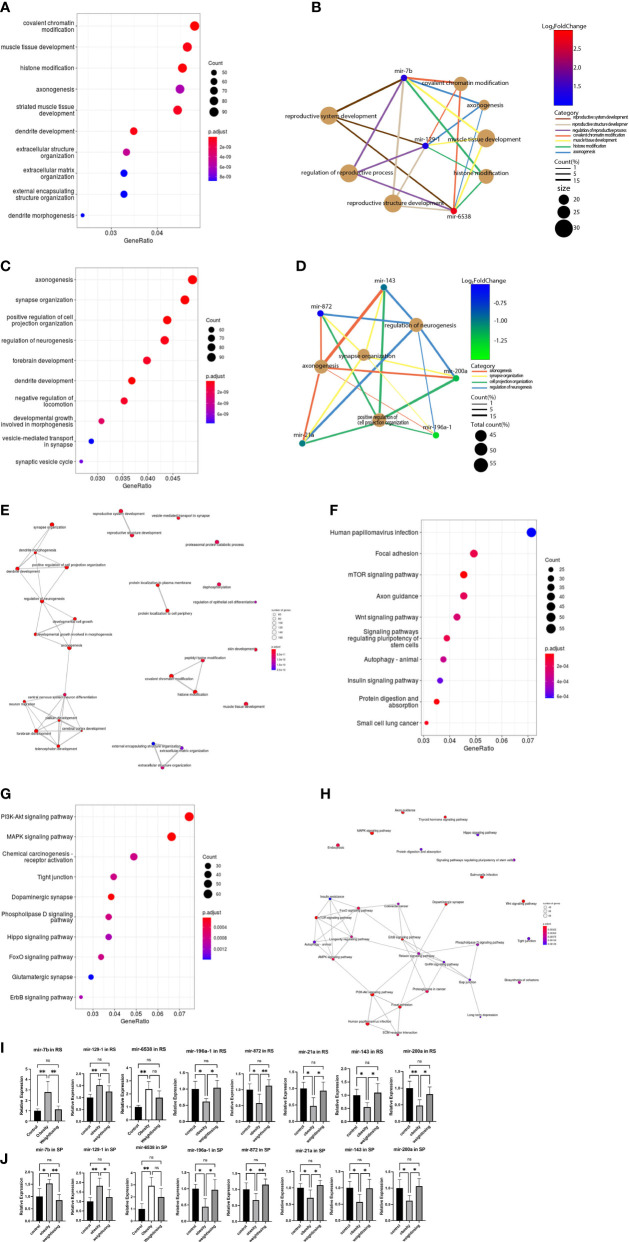
GO analysis and KEGG pathway analysis of target genes of consistently differentially expressed miRNAs revealed significantly enriched biological process GO terms and signaling pathways. **(A)**, The top 10 significantly enriched GO terms in the biological process (BP) category associated with target genes of 3 consistently upregulated miRNAs. **(B)**, Gene-concept network for the 3 consistently upregulated miRNAs with the associated top 4 and reproductive system-enriched BP terms as a net. **(C)**, The top 10 significantly enriched BP GO terms associated with target genes of 5 consistently downregulated miRNAs. **(D)**, Gene-concept network for the 5 consistently downregulated miRNAs with the related top 4 enriched BP terms as a net. **(E)**, GO-Tree network analysis based on the interaction relationship of enriched BP terms. **(F)**, The top 10 enriched signaling pathways associated with target genes of 3 consistently upregulated miRNAs. **(G)**, The top 10 enriched signaling pathways associated with 5 consistently downregulated miRNAs. **(H)**, Pathway-act network analysis illustrated mutual interactions between pathway terms. **(I)**, The relative expression levels of 3 consistently upregulated miRNAs and 5 downregulated miRNAs in round spermatids were validated by real-time PCR. **(J)**, The relative expression levels of 3 consistently upregulated miRNAs and 5 downregulated miRNAs in epididymal spermatozoa were validated by real-time PCR. One-way ANOVA was used to compare the differences among the three groups. Control: n=5, obesity: n=5, exercise: n=5. ns: no significant, p value > 0.05, *: P < 0.05, **: P < 0.01. Data are expressed as the mean ± SD.

To elicit the biological implications of consistently differentially expressed miRNAs in the etiology of impaired male fertility, we conducted GO functional annotation for the target genes of differentially expressed miRNAs. For BPs, the top five enriched GO terms associated with upregulated mRNAs were covalent chromatin modification, muscle tissue development, histone modification, axonogenesis, and striated muscle tissue development (**Figure 3A**). The 3 consistently upregulated genes were enriched in GO terms related to chromatin modification, neuromuscular development, and reproductive system development (**Figure 3B**). The top five enriched GO terms associated with downregulated miRNAs included anoxogenesis, synapse organization, positive regulation of cell projection organization, regulation of neurogenesis, and forebrain development (**Figure 3C**). The 5 consistently downregulated genes were enriched in GO terms related to nervous system development (**Figure 3D**).

To further understand the core BP associated with male fertility and offspring development, GO-Tree analysis of upregulated and downregulated BP GO terms was performed based on their subordinate and interaction relationships (**Figure 3E**). The network indicates that BPs, including reproductive system development and reproductive structure development, may play a key role in paired male fertility. These BPs, such as central nervous system neuron differentiation, neuron migration, pallium development, cerebral cortex development and forebrain development, may play an important role in the development of the nervous system in offspring. BPs, such as covalent chromatin modification, histone modification, and peptidyl-lysine modification, may be involved in epigenetic inheritance across generations.

To indicate the signaling pathways associated with target genes of consistently differentially expressed miRNAs, KEGG pathway analysis of target genes was performed. The top five enriched pathways associated with upregulated miRNAs were human papillomavirus infection, focal adhesion, mTOR signaling pathway, axon guidance, and Wnt signaling pathway (**Figure 3F**). For downregulated miRNAs, the top five enriched pathways were the PI3K-Akt signaling pathway, MAPK signaling pathway, chemical carcinogenesis-receptor activation, tight junction, and dopaminergic synapse (**Figure 3G**).

A pathway-act network was explored to further investigate the mutual interactions of pathways and to obtain the hub pathways that may play a vital role in male fertility and offspring development (**Figure 3H**). The relaxin signaling pathway, FoxO signaling pathway, ErbB signaling pathway, longevity regulating pathway, and PI3K-Akt signaling pathway were the top five pathways showing the most interactions with other surrounding pathways. The relaxin signaling pathway is closely related to the reproductive system. The FoxO signaling pathway is involved in glucose metabolism. The longevity-regulating pathway includes the insulin signaling pathway, mTOR pathway, and AMPK signaling pathway coregulating glucose metabolism.

The consistently differentially expressed miRNAs and P values were further verified by quantitative real-time PCR. The results indicated that mir-6538, mir-129-1, and mir-7b were upregulated (**Figure 3I**) and that mir-196a-1, mir-872, mir-21a, mir-143, and mir-200a were decreased (**Figure 3J**) in the obesity and control groups in RS and SP. These results are consistent with the RNA-Seq profile data. In addition, physical exercise of obese mice could restore these changes induced by a high-fat diet in RS and SP (**Figures 3I, J**).

## Discussion

Our research found strong, consistent evidence that a high‐fat diet is detrimental for male fertility. Compared with controls, obese males fed a high‐fat diet exhibit adverse changes in their reproductive system, including reduced testes and seminal vesicles relative to body size, and they produce fewer sperm and a lower proportion of motile sperm. In addition, obese males present reproductive hormone disorders. More importantly, males fed a high‐fat diet have impaired sperm function and reduced reproductive success. Accordingly, physical exercise could partially or even completely offset the adverse effects of obesity, but it needs to have the prerequisites of body weight and body fat rate significantly below the normal level. It is a serious issue which is worth discussing to explore its reason, and among these, the changes of microRNA in round spermatids and spermatozoa may explain the effects of obesity and physical exercise on sperm function.

Previous human studies have little evidence supporting that increased BMI is related to reduced standard semen assessment traits (5, 6, 28), and yet our present results demonstrated that all measures of sperm quantity and quality are negatively impacted when obesity is experimentally induced with a high‐fat diet, which was consistent with a high-quality meta-analysis including 52 animal studies (29). Moreover, the magnitude of the negative effects of a high‐fat diet on sperm traits was increased when the difference in body mass of control and treatment animals was higher (29). Based on that, the outcome of male mating and pregnancy could be consequently affected by a high-fat diet; this is corroborated by our reproductive experiment, and importantly, the damage rates of mating and pregnancy were more likely to be amplified by long-term high-fat diet treatment. In addition to impaired sperm quantity and quality, male behavior, lack of libido or inability to mount might partly explain the reduced mating success. Abnormal male behavior might be attributed to reproductive hormone disorder, reduced testosterone and elevated estradiol. Apart from the influence of behavioral factors on pregnancy, how can the influence of obesity on reduced fertilization success be considered? The answer can be found in the IVF population. Several population studies have shown that the clinical pregnancy rate of IVF in couples with obese husbands is lower, which may be related to the sperm itself, such as delayed capacitation and acrosome reaction, rather than the number or concentration of spermatozoa (30). The higher proportion of obese men in infertile couples who choose intracytoplasmic sperm injection (ICSI) can also explain this finding because ICSI can prevent clinical pregnancy failure caused by insufficient sperm quantity, poor motility and delayed capacitation and acrosome reaction in IVF.

Do epigenetic changes or oxidative stress damage in spermatozoa caused by obesity affect clinical pregnancy? A study showed that among couples undergoing intracytoplasmic sperm injection, the odds of live births for couples with obese male partners were 84% lower than the odds in couples with men with normal BMI (31). Another study also demonstrated that overweight men with nonobstructive azoospermia have worse pregnancy outcomes after testicular sperm aspiration (TESA) and ICSI (32). Data from investigating sperm epigenetic molecular status and embryo morphokinetics show the negative impacts of obesity on motile spermatozoa molecular composition and the possible risk of disturbing early embryonic cell cycle kinetics in the context of paternal obesity (33). Even so, there is conflicting evidence. A retrospective study supported no evidence for a relationship between male BMI and treatment outcomes of ICSI (34). In patients with nonobstructive azoospermia who underwent TESA-ICSI, a Chinese study showed that embryo quality and clinical outcomes were not influenced by high BMI levels (35). Therefore, this question warrants further investigation. Significantly, in our present study, it is clear that obesity causes a decreased pregnancy success rate in mice, which is effectively improved by physical exercise.

The next concept is whether a high-fat diet could affect spermatozoa by activating small RNA molecules. There is a widely accepted view that miRNAs may act as fine-tuners of large gene networks (36–38). miRNAs ensure accurate gene expression in the adult testis by keeping levels within the required thresholds, thus playing a crucial role in testis homeostasis (39). In our present study, we used small RNA-Seq data to reveal that the round spermatids and epididymal spermatozoa expression patterns of miRNAs are significantly altered in the obesity group compared with the controls. For the first time, we found several miRNAs that were consistently differentially expressed both in RS and SP between the two groups. The target genes regulated by these miRNAs were found to be involved in metabolic regulation, nervous system development regulation and chromosome modification according to GO and KEGG analyses. Why were these miRNAs consistently differentially expressed? miRNAs are involved in the regulation of gene expression during the postmeiotic stages of spermatogenesis. Specifically, miRNAs can repress the transcription of transition protein 2 (40), a marker for round spermatids, suggesting that miRNA functions affect postmeiotic germ cells. Translin (also known as testis-brain RNA-binding protein) has been demonstrated to bind to miRNAs and thus increase the *in vivo* stability of miRNAs (28). Furthermore, elongated spermatids exhibit abnormal morphology and motility, and consequently, male infertility occurs in Dicer1-knockout mice, indicating that both Dicer1 and miRNAs play crucial roles in proper differentiation during spermatogenesis. Rao et al. (29) demonstrated that the Wilms’ tumor 1 (WT1) transcription factor, which could be repressed by miRNAs, plays an essential role in the control of germ cell survival and spermatogenesis. WT1 knockdown mice suffered from increased germ cell apoptosis, a loss of the adherent junction complex between germ cells and Sertoli cells, and impaired fertility. In addition, prevailing data demonstrate that proteins and miRNAs in the epididymal fluid associated with post testicular sperm maturation are transferred to the sperm by EVs (30). These differentially expressed miRNAs due to the high-fat diet treatment may be contained in extracellular vesicles and transferred to mature sperm through the Sertoli cells of the testis and epididymal fluid. Taken together, these studies further indicate that normal miRNA biogenesis is required for round spermatids to become epididymal spermatozoa.

Interestingly, in our present research, some altered miRNAs induced by high-fat diet are recovered *via* physical exercise including 3 consistently upregulated miRNAs (miR-6538, miR-129-1, and miR-7b) and 5 consistently downregulated (miR-143, miR-872, miR-21a, miR-196a-1, and miR-200a), revealing that physical exercise could participate remodeling of reproductive system development and regulation of reproductive processes. A previous study showed that Ppp2r2b, Rgs8 and Atp1b2, as the target genes of mir-7b, showed a complex interaction within a biological process leading to sperm dysfunction upon exposure to fluorosis (41). MiR-7b interacts with the 3’ untranslated region of the immediate-early gene Fos mRNA and inhibits Fos translation. It shows rapid induction and dimerization to form transcription factor activator protein 1 (AP-1) after stress stimulation, such as diet alteration, which is known as a pivotal regulator of major biological events, such as cell proliferation, differentiation, organogenesis, memory formation and apoptosis, in germ cells to regulate fertility (31). Increased miR-129 was previously proven to significantly reduce the expression of the mitochondrial Cox family, together with that of MyHC I, and knockdown of miR-129 conversely increased the expression of cox genes and MyHC I. Mature spermatozoa are rich in mitochondria, especially in the tail sheath, and the inhibition of mitochondrial genes by the mir-129 family significantly affects sperm motility (32). Additionally, it is important to note that the altered expression profiling of miRNAs in round spermatids and epididymal spermatozoa induced by a high-fat diet in our present data could affect the adult health condition in offspring *via* patterns that are not yet clear. It is well known that the nutritional status of the father affects the development of the offspring. In rodent studies, increased anxiety-like behavior was documented in offspring sired by males that consumed a high-fat diet (33). In humans, paternal BMI was found to be inversely associated with child IQ (34). It is certain that physical exercise to lose weight can partly counteract the effect of an obese father on the metabolic abnormalities of the offspring, mainly through increasing the absorption and utilization of glucose by skeletal muscle in the offspring (35, 36). These results are consistent with our findings that these altered miRNA profiles are involved in metabolic regulation, nervous system development regulation and chromosome modification according to GO and KEGG analyses, suggesting their potential additional adverse effects and involvement in the intergenerational transmission of obesity through gametes.

Epigenetic modifications, including functional sncRNAs in spermatogenesis, are directly involved in the etiopathology of adult diseases transmitted by sperm. To determine whether sperm ncRNA is the determinant of intergenerational heredity, Grandjean et al. microinjected ncRNA of obese mice spermatozoa into fertilized eggs from normal parents. It was found that the offspring had more weight gain and impaired glucose tolerance and insulin resistance in adulthood, although they kept a normal diet (37). This is consistent with the results of Fullston et al. (38). Chen et al. microinjected tRFs (tRNA-derived fragment, one of sncRNAs) extracted from the spermatozoa of obese male mice into normal fertilized eggs fed a normal diet and found that in adulthood, they showed impaired glucose tolerance but no insulin resistance (15). The above evidence supported that sncRNAs can undertake part of the transmission from father to offspring. Our present findings reveal the upregulated levels of miR-143, miR-872, miR-200a, miR-21 and miR-196a in obese mice rescued by physical exercise implying that physical exercise can restore the possibility of disease transmission from father to offspring. It is well known that miR-143 can control Notch receptor expression by binding and releasing lncRNAs. In addition, overexpression of miR-143 can also reduce cell proliferation and induce neuronal differentiation. MiR-143-mediated lncRNA function in the developing mouse cortex leads to an expansion of PAX6+ RGCs. In addition, miR-872 can directly target the protein SOD-1, a copper/zinc superoxide dismutase, and its overexpression in neurons is associated with increased cell death through apoptosis (39). mir-200a regulates neural induction by targeting zinc finger E-box-binding homeobox (ZEB) transcription factors, allowing embryonic stem cells to differentiate into neuroectodermal precursors rather than epidermal cells (41). Inducing the expression of mir-21 can improve cognitive function in patients with Alzheimer’s disease, thus reflecting its active participation in neuromodulation processes, integrating cognitive signaling pathways, and strongly affecting cognitive development (42). miR-196a can directly interact with the IκBα 3’-UTR to inhibit IκBα expression and subsequently promote the activation of NF-κB, thereby promoting neuronal proliferation and inhibiting apoptosis *in vivo* and *in vitro*, thereby regulating neural development (43). These findings support that miRNAs contribute to the expansion of the cerebral cortex and the development of the nervous system (44), and it is reasonable to speculate that these functional microRNAs affect the nervous system development of offspring across generations to offspring through sperm according to our present study.

In this study, we also found that Sin3a and Rnf40, which are targeted by mir-7b, participate in the regulation of histone modification. MiR-129-5p can regulate HDACi, which is responsible for histone deacetylase inhibitors (45). In addition, Per1 is targeted by miR-6538, and its expression can be regulated by HDACi (46). Therefore, these microRNAs coordinate histone modifications. In mammalian sperm, the majority of nucleosomes are replaced with protamines to facilitate the compaction of the paternal genome (47). Nevertheless, a small percentage of nucleosomes and their associated histone modifications are retained, thereby forming a potential platform for the intergenerational transmission of regulatory states (48, 49). Both active (H3K4me2 and H3K4me3) and repressive (H3K27me3, H3K9me3 and H4K20me3) histone modifications have been detected in mammalian sperm. For example, in humans, H3K9me3-marked and H4K20me3-marked nucleosomes are transmitted through the sperm into the zygote, where they participate in the build-up of constitutive heterochromatin (50). Notably, these changes promoted developmental defects in the offspring and were transmitted across three generations.

In summary, this study clearly shows that obesity due to paternal diet can cause significant impairment of many sperm function parameters, including decreased motility and delayed capacitation and acrosome reaction. When the weight and body fat rate after physical exercise are lower than normal, the adverse effects of obesity could be offset. Small noncoding RNAs in spermatogenic cells may play an important role in the effects of obesity and physical exercise on male fertility and offspring development. Our findings provide new evidence of the relationship between obesity, physical exercise, male infertility and offspring development.

## Data availability statement

The original contributions presented in the study are publicly available. This data can be found here: https://www.ncbi.nlm.nih.gov/sra/PRJNA904533.

## Ethics statement

The animal study was reviewed and approved by the animal ethics committee of Shanghai Model Organisms.

## Author contributions

HH, XL devised the study concept and design. TL and SZ drafted the manuscript, completed the experiment, and collected data. YZ provided support in experimental technology. LW was responsible for the statistical analysis and quality control. All authors agreed with the final version of the article.

## Funding

This research was supported by the National Natural Science Foundation of China (82088102, 82171687, 32071131), CAMS Innovation Fund for Medical Sciences (2019-I2M-5-064), National Natural Science Funds (82192873), Collaborative Innovation Program of Shanghai Municipal Health Commission (2020CXJQ01), Clinical Research Plan of SHDC (SHDC2020CR1008A) and Shanghai Frontiers Science Research Base of Reproduction and Development. Ethics approval was obtained from Shanghai Model Organisms review board on August 20th, 2019. (IACUC 2019-0016)

## Conflict of interest

The authors declare that the research was conducted in the absence of any commercial or financial relationships that could be construed as a potential conflict of interest.

## Publisher’s note

All claims expressed in this article are solely those of the authors and do not necessarily represent those of their affiliated organizations, or those of the publisher, the editors and the reviewers. Any product that may be evaluated in this article, or claim that may be made by its manufacturer, is not guaranteed or endorsed by the publisher.
