# Supplementary material for: Small RNA perspective of physical exercise-related improvement of male reproductive dysfunction due to obesity
Source: Front Endocrinol (Lausanne). 2022 Dec 2;13:1038449. doi: 10.3389/fendo.2022.1038449 (PMC9756842; doi:10.3389/fendo.2022.1038449)
Supplement: Supplementary file 1 [file DataSheet_1.docx]

Supplementary

Supplementary Table 1 The primers of microRNAs for RT-qPCR

| microRNA | Stemloop | Forward primers | Reverse primers |
| --- | --- | --- | --- |
| mir-7b-5p | GTCGTATCCAGTGCAGGGTC CGAGGTATTCGCACTGGATACGACAACAAC | TGGAAGACTTGTGATTTT | GCAGGGTCCGAGGTATTC |
| mir-129-1-3p | GTCGTATCCAGTGCAGGGTC CGAGGTATTCGCACTGGATACGACATACTT | AAGCCCTTACCCCAAA | GCAGGGTCCGAGGTATTC |
| mir-6538 | GTCGTATCCAGTGCAGGGTC CGAGGTATTCGCACTGGATACGACCGCCGC | CGCGGGCUCCGGG | GCAGGGTCCGAGGTATTC |
| mir-143-3p | GTCGTATCCAGTGCAGGGTC CGAGGTATTCGCACTGGATACGACTGATAT | GAGCTACAGTGCTTC | GCAGGGTCCGAGGTATTC |
| mir-872-3p | GTCGTATCCAGTGCAGGGTC CGAGGTATTCGCACTGGATACGACAGGAGG | TGAACTATTGCAGTAG | GCAGGGTCCGAGGTATTC |
| mir-21a-5p | GTCGTATCCAGTGCAGGGTC CGAGGTATTCGCACTGGATACGACTAGCTT | TCAACATCAGTCTGAT | GCAGGGTCCGAGGTATTC |
| mir-196a-1-3p | GTCGTATCCAGTGCAGGGTC CGAGGTATTCGCACTGGATACGACATACGG | CAACGACATCAAACCA | GCAGGGTCCGAGGTATTC |
| mir-200a-3p | GTCGTATCCAGTGCAGGGTC CGAGGTATTCGCACTGGATACGACTAACAC | ACATCGTTACCAGACA | GCAGGGTCCGAGGTATTC |


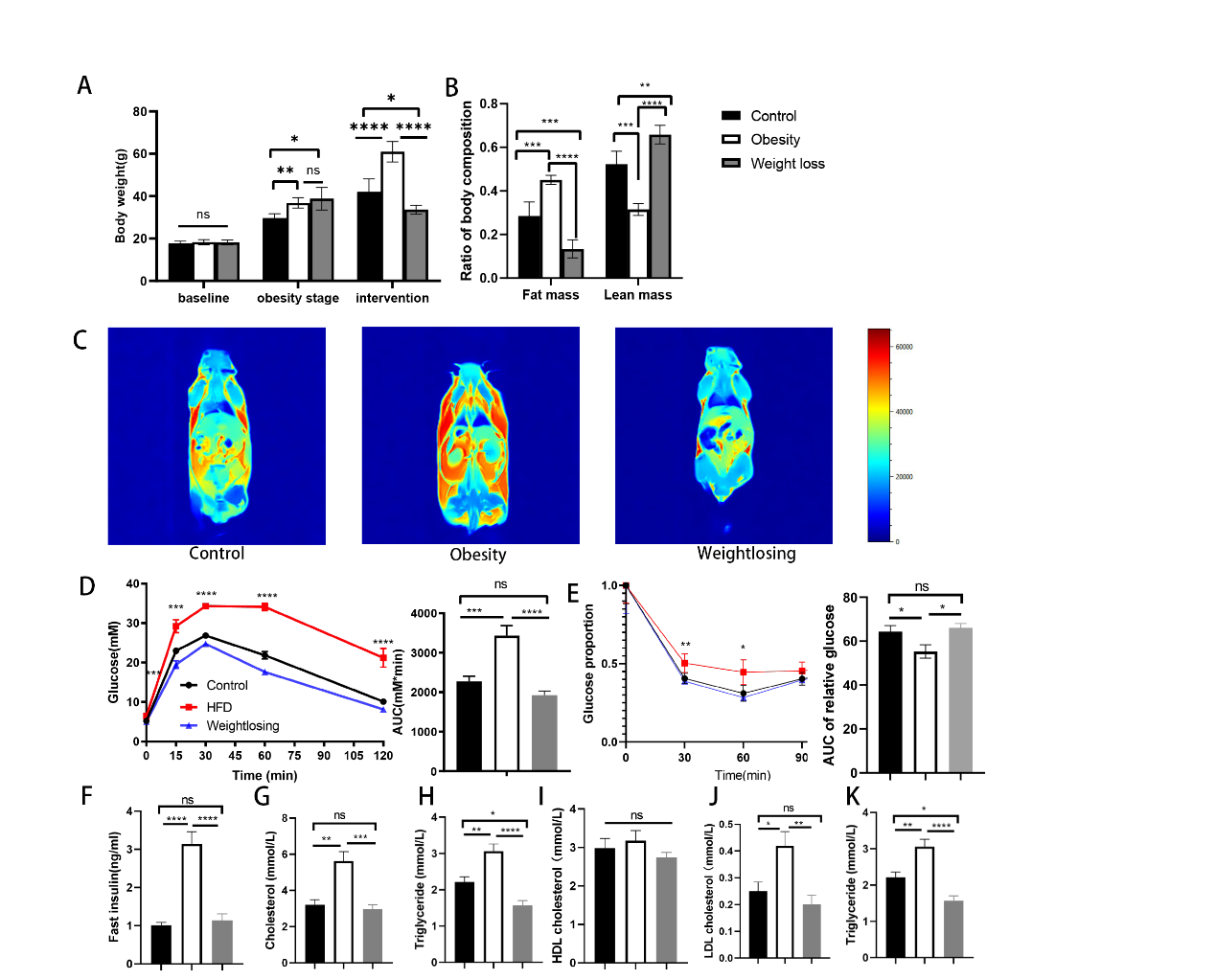


**Supplementary Figure 1 Effects of physical exercise intervention on body weight, body components, and glucose and lipid metabolism.** A, Body weight of the three groups at different time points (baseline, when the obesity model was completed, when the physical exercise intervention was completed). B, The ratio of fat mass or lean mass to body weight in the three groups. C, Representative body images. D, Glucose levels during GTT and the area under the curve. After an overnight fast, glucose (mM) levels were measured in the fasting state and 15, 30, 60, and 120 min after oral administration of glucose solution via gavage (0.75 g glucose/kg). E, Glucose levels during ITT. The glucose (mM) levels were measured in the fasting state and 30, 60, 90, and 120 min after intraperitoneal injection of insulin (1.0 U insulin/kg). F, Serum insulin (ng/ml) levels were measured after an overnight fast. G, Serum cholesterol (mM), H, triglyceride (mM), I, high-density lipoprotein cholesterol (mM), J, low-density lipoprotein cholesterol (mM), K, nonesterified fatty acid (mM) levels were randomly measured. One-way ANOVA was used to compare the differences among the three groups. Control: n=5, obesity: n=6, exercise: n=5. ns: P>0.05, *: P<0.05, **: P<0.01, ***: P<0.001, ****: P<0.0001. Data are expressed as the mean ± SEM


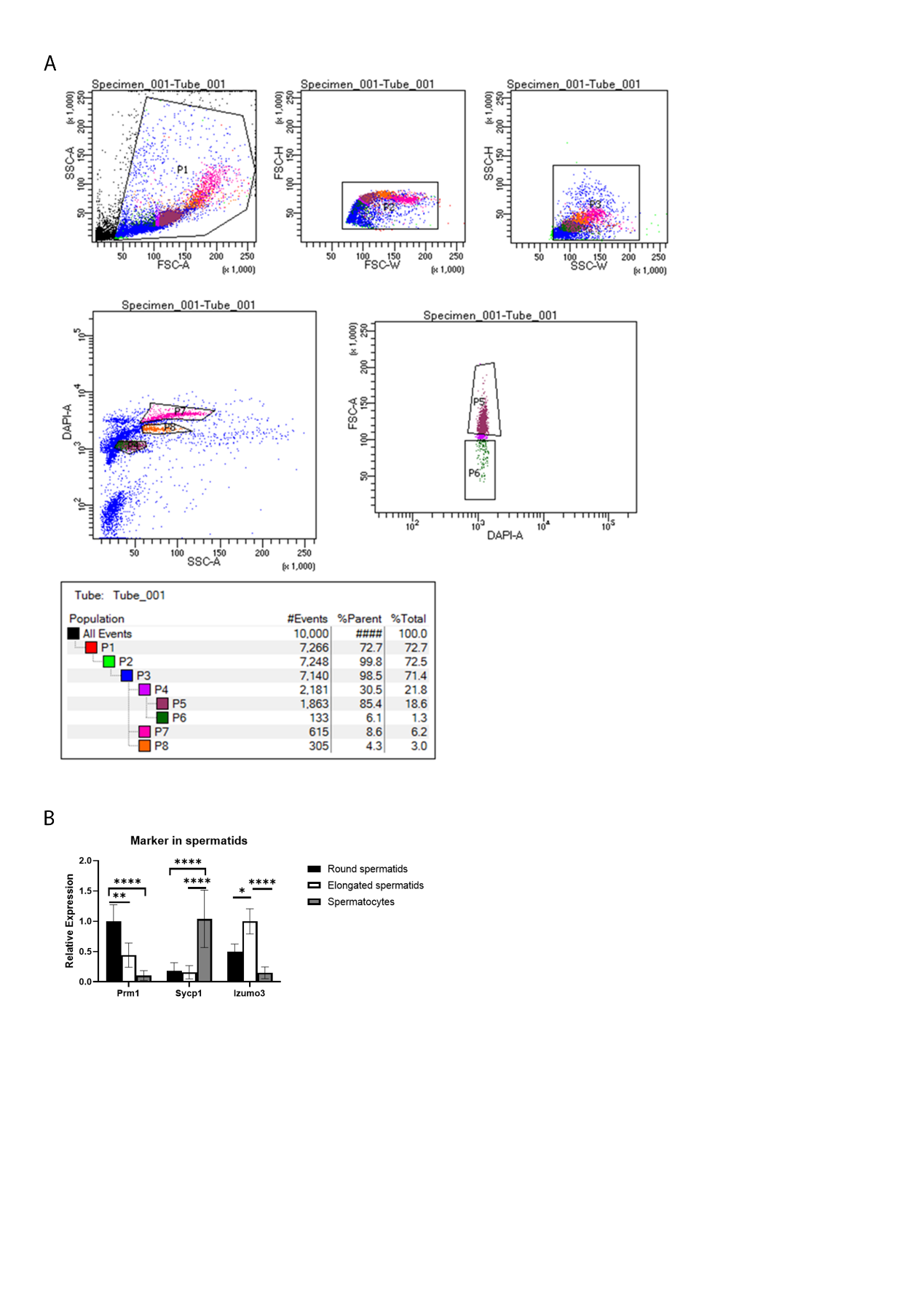


**Supplementary Figure 2 The isolation and validation of round spermatids.** A, Representative sorting gates applied for purifying round spermatids from the testis using cell sorting by flow cytometry. B, Validation of round spermatids with markers by PCR.
